# Supplementary material for: The causal relationship between the human gut microbiota and pyogenic arthritis: a Mendelian randomization study
Source: Front Cell Infect Microbiol. 2024 Nov 26;14:1452480. doi: 10.3389/fcimb.2024.1452480 (PMC11629706; doi:10.3389/fcimb.2024.1452480)
Supplement: Supplementary file 1 [file DataSheet1.zip › mendelian test/ebi-a-GCST90027679.csv_forest.pdf]

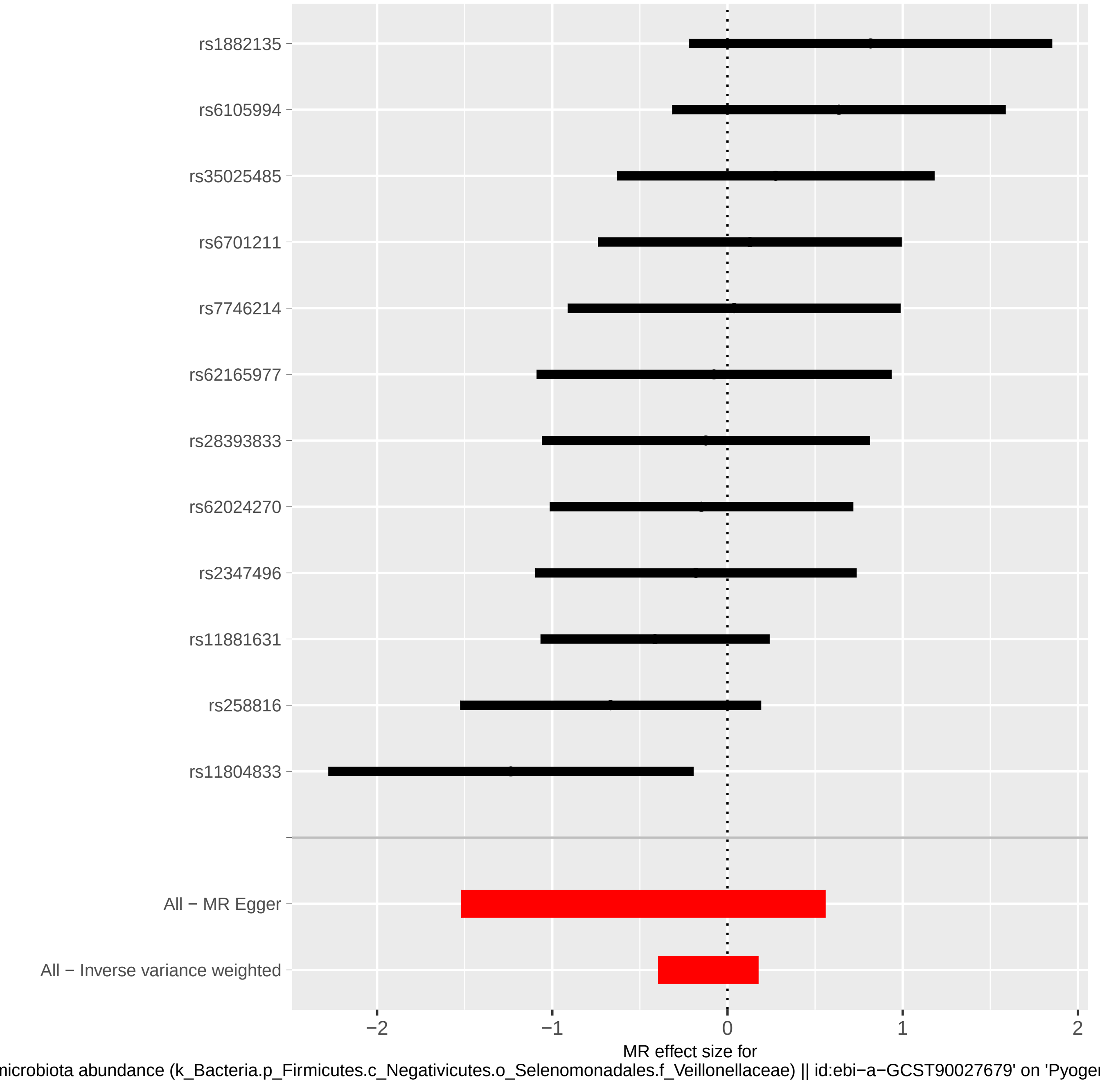

rs1882135
rs6105994
rs35025485
rs6701211
rs7746214
rs62165977
rs28393833
rs62024270
rs2347496
rs11881631
rs258816
rs11804833
All − MR Egger
All − Inverse variance weighted
−2
−1
0
1
2
MR effect size for
microbiota abundance (k_Bacteria.p_Firmicutes.c_Negativicutes.o_Selenomonadales.f_Veillonellaceae) || id:ebi−a−GCST90027679' on 'Pyoger
